# Young carers’ experiences of services and support: What is helpful and how can support be improved?

**DOI:** 10.1371/journal.pone.0300551

**Published:** 2024-03-29

**Authors:** Madeleine Stevens, Nicola Brimblecombe, Sara Gowen, Robin Skyer, Jo Moriarty

**Affiliations:** 1 Care Policy and Evaluation Centre, London School of Economics and Political Science, London, United Kingdom; 2 Sheffield Young Carers, Sheffield, South Yorkshire, United Kingdom; 3 NIHR Health & Social Care Workforce Research Unit, King’s College London, London, United Kingdom; Universidad Internacional de La Rioja, SPAIN

## Abstract

Globally, many children and young people provide support to family members who have poor physical or mental health, are disabled, or misuse drugs and alcohol. These young carers are at higher risk of poorer education, employment, health, and social participation outcomes compared to their peers without caring responsibilities. In the UK, awareness of the challenges faced by young carers, and a framework of their legal rights, are relatively well-developed. However, it is unclear how support can most effectively be provided. Taking a qualitative approach we explored experiences and views of young carers (aged 9–25), conducting focus groups or interviews with 133 young carers and 17 parent care recipients. We explored what aspects of services and support are seen as helpful, valued, and acceptable to young people, and what could be improved. A reflexive, thematic analysis was conducted. Valued support came from: young carers groups (including peer support), school-based and mental health support, and support for the care recipient. Helpful aspects of support included someone who listens and understands, and can be trusted not to break confidentiality; involving the young person in information, decision-making and planning (sometimes including regarding the care recipient); and finding and linking to other services. There was a difficult balance for practitioners between being perceived as proactive, persistent or intrusive when offering support to a young carer, but it was important to allow opportunities for young carers, and those they care for, to change their minds about when and whether to access support. Many interactions were perceived as unhelpful or threatening to the family, and there was often not enough of the type of support that was valued. Sharing of positive experiences can be beneficial for both people seeking support and those delivering it; key messages on what is helpful from the perspective of young carers can help support and shape practice approaches.

## Introduction

Previous research has described the impacts on young people of providing unpaid care to a family member, including negative impacts, compared to peers without caring responsibilities, on health and wellbeing [1–4], education [1, 5, 6], and employment prospects [1, 7, 8]. Research has also suggested some positive impacts of caring such as closer relationships with parents, feelings of preparedness for life, and fostering of empathy and compassion [1]. Awareness of the potential difficulties faced by young carers is relatively high in the UK compared to other countries and the UK has a relatively well-developed policy framework in relation to young carers [9]. Young carers and the people they support have been given explicit rights to support in UK policy; professionals have a duty to identify and support young carers and their families [4, 9–11]. However, it appears that the effects of this legislation have been limited [12] and it has been argued that clearer guidance is needed [13]. Costs associated with negative outcomes of young caring are high and therefore there is potential for services to be cost-effective if the right kind of support is put in at the right time [14].

To improve practice in how best to identify and support young carers, there needs to be greater understanding of what type of support young carers and their parents find useful, why some funded support is not seen as useful, and, importantly, why some offers of support are turned down. This is currently a research gap; little is known about what young carers find helpful and few studies have prioritised the views of young carers. Some groups of relatively so-called ‘hidden’ young carers (those not identified by services, and/or who may not think of themselves as carers), including those from ethnic minorities, are known to be particularly unlikely to access services [11]. Recent findings have referred to some of the problems with available support [5, 15], and young carers have been shown to value opportunities for respite and fun activities that give them a break [16, 17]. Previous studies which ascertained young carers’ thoughts on what support would best support them indicate that better support for the person they care for is the most important [1, 16]. This has led to the identification of ‘whole family support’, that takes into account all family members’ needs, and how they are interrelated, as a useful approach to supporting young carers [18–20].

Few studies have looked at the support provided to young carers in schools [21] but in one small study an ambivalence about disclosing a ‘young carer’ status, and therefore accessing support, was found, as well as frustration with teachers’ lack of understanding about the difficulties faced by young carers [22]. An evaluation of the Young Carers in Schools Programme in England, aimed at improving schools’ ability to identify and support young carers, found varied degrees of implementation [23]. The school-based practitioners surveyed, mainly those with a responsibility for young carers, were positive about the scheme, and reported increased identification of young carers, more referrals to support, and that young carers in schools benefitted from group activities and peer support [23]. Other research also found positive effects of peer support [24, 25]. Young carers at school or college have identified areas where they would like more support, including difficulties making friends when peers do not understand their caring role [26]; difficulties keeping up with schoolwork or homework [27]; and bullying [6].

In the UK, support available to young carers has been shown to be inconsistent across the country [28], and studies in both the UK and Australia have shown how lack of time due to caring responsibilities, and lack of understanding from professionals, have been barriers to young carers engaging with available support [29, 30].

There is, then, research which looks at unmet need among young carers and research exploring particular programmes or approaches that set out to provide solutions, but there is a lack of studies taking a holistic approach to looking at what is found to be useful, and a lack of investigation of these issues from the perspective of young carers themselves. The current study set out to address this gap, by engaging with a large number of young carers in England usingan in-depth, qualitative approach. We aimed to explore young carers’ experiences with support received, across all types of service, including health and social care, education and the voluntary and community sectors, and both formal support (usually provided by professionals paid for their work) and informal support (for example from friends and family). We aimed to identify what aspects of support were found useful, and what stops existing help from being helpful. Exploring these issues in-depth can help inform practice in meeting the aims of policy to promote effective support for young carers and the people they care for. The findings will have relevance beyond the UK for countries developing policy and practice towards young carers.

In our paper, as elsewhere, we define young carers as children and young people who look after, or give help or support to, a family member who needs help because of their long-term physical or mental ill health, substance misuse, disability, and/or problems related to old age [31]. Young carers are generally considered those aged under 16 while the term young adult carers is used to refer to those aged 16–25. In this paper we use the term young carers to refer to both age groups.

## Methods

Because of the research gap around young carers’ experiences of services we took a qualitative, exploratory approach to look at what types, components or features of services and other support are seen as helpful, valued, and acceptable to young people who look after someone at home and the people they support and, conversely, what is found to be less or unhelpful. The rationale for the approach was that we wanted to hear from those with ‘lived experience’ of being young carers, as well as those they care for [32], since this is a gap in the research, one that we felt was essential to fill. The perspectives of young carers in particular, about what is helpful or unhelpful in supporting them and the people they care for, is necessary to inform policy and practice and to contribute to understanding gaps between policy and practice. We used qualitative methods, rather than a survey, for example, because we considered, supported by discussions with advisors and our previous work, that the issues we wished to find out about required discussion and development of shared understandings; we did not feel that it would be easy to adequately interpret survey responses to questions on the topic of good and bad services. An exploratory, rather than confirmatory approach was needed because not enough is known about young carers’ attitudes towards, and experiences of, receiving and not receiving services [32]. We also understood that a certain amount of trust needed to be developed in order for views and experiences to be shared. To this end we worked closely with a range of advisors including an advisory group of young carers to develop methods that would be inclusive, put young people at ease, and allow participants to contribute in a variety of ways. We also worked closely with practitioners who work with young carers.

### Sample

Young carers organisations in four localities around England helped us recruit young carers to participate. The areas were chosen to be contrasting in terms of ethnicity and rurality; all contained areas of high deprivation. We drew on their extensive outreach work to include particularly marginalised groups of young carers, for example those living in rural areas and those caring for parents with stigmatised conditions such as severe mental illness. The inclusion criteria were young carers aged between 9 and 25 and providing unpaid care. Within these criteria we aimed to be as inclusive as we could and involve young people from a wide variety of backgrounds, particularly those who are least often heard [33]. In addition, the collaborating young carers organisations invited parents who were cared for by a young carer to take part in a focus group or interview, leading to two parent carer focus groups and two interviews.

In total we spoke with 150 participants. 17 of these were adult care recipients. The young carers (aged between 9 and 25) mainly provided care for a parent, but some cared for a sibling.

We did not ask participants to provide individual-level demographic data as it was agreed with advisors and collaborators that people could be wary of formally sharing such data with strangers (the researchers) and there could be negative impacts on rapport, trust and the sharing of experiences. Nevertheless, the collaborating organisations provided ages of attendees and in some cases had been specifically asked to recruit young carers of people with mental health difficulties; in many cases young people voluntarily shared information as part of introductions in groups; we are therefore able to give an overview of demographics in most cases (Table 1).

**Table 1:** Study participant descriptives (where known).

Descriptor	n
Young carer research participant	133
Age in years	
9–11	40
12–15	57
16–25	36
Caring for parent	46
Caring for a sibling	25
Caring for another relative	2
Condition of person cared for by young carer participants	
Mental ill health	26
Physical ill health	19
Substance misuse	8
Other (including dementia, neurodiversity, learning disability)	5
Parent care recipient participant	17

### Data collection

Data collection methods were developed and discussed in collaboration with our young carer advisory group, study steering group, and participating young carer organisations, and were also informed by previous research [34]. Data collection was primarily by focus group; this decision was also informed by previous research finding that focus groups can be more effective than interviews and questionnaires where people may not have given the issues to be discussed much previous consideration, and because opinions are not formed in isolation [35]. We carried out 19 focus groups with young carers. Young carers were usually in groups with people of a similar age, for example, primary school age, secondary school age and 16+. Two focus groups were carried out with parents who were recipients of care from a young carer.

The focus group format can be less threatening for some [36], and research has suggested that younger participants in particular may respond better in a group setting [37]. However, we offered young carers and care recipients who couldn’t or preferred not to attend focus groups an opportunity to be interviewed instead, in person or by phone/online. Two interviews were carried out in-person with care recipients, and eight interviews were carried by phone or online with young carers.

At least one practitioner with expertise in working with young carers attended each group. Many participants were already acquainted with the attending practitioners, who had earned their trust over time. In the sessions participants had the opportunity to share their views both with and without these practitioners overhearing. Each session was led by two researchers. The two university-based researchers were experienced in working with young people, including young carers, in a variety of contexts; they were not known to any of the participants in advance of the study. The third researcher was a social care practitioner experienced in working with young carers and their families. This researcher was acquainted with some of the participants in one location.

The detailed plans and topic guides for the focus groups and interviews were developed in collaboration with our young carer and practitioner advisors (see above). In the focus groups, following fun ice-breaker activities we began by working as a group to develop a list of all the different types of services young carers (or care recipients) come into contact with. Some discussion about these services took place during the making of the list, which was done on a flipchart that the whole group would see. Once we had the long list of services, we then took participants up to the flipchart, usually in pairs. Each participant had their own set of coloured stickers and during the discussions at the flip chart they were asked to rate services they had been in contact with, using stickers of different colours. The colours indicated whether the service or support had been good, ok but could be better, or bad.

This rating exercise then led to discussion with the pair at the flipchart, or sometimes a single person, of what was helpful and unhelpful about services. We could ask, ‘so you gave this one a bad rating–could you tell me about that?’ The chart was annotated with key points, and the researcher at the flipchart also took notes, especially where information was personal and not appropriate to record on the flipchart. When points were added to the flipchart, this was discussed with the participant (‘shall I write that down’?). While some participants were at the flipchart, the remainder carried on with the next activity.

The sessions were audio-recorded on two different recorders in different parts of the room and where possible (where voices could be distinguished sufficiently clearly) transcribed. Long sections of the meetings were not transcribable due to overlapping voices and ambient noise, and the researcher facilitators took extensive notes to record participants’ comments. Researchers reviewed and added to their notes at the end of the sessions, while fresh in the mind.

Later sections of the group activities involved an exercise in blue skies thinking’ where participants imagined their ideal services, and there was a section where participants wrote on paper ‘bricks’ to represent barriers to accessing services. The findings from these parts of the research are written up separately [38].

At the end of the focus group the researchers summarised their preliminary understanding of the key messages they had heard from participants during the session. Each researcher read out a set of statements summarising the key points they had learnt, and participants were invited to hold up coloured cards to indicate their degree of agreement with the statement. Green indicated ‘agree’, red ‘disagree’ and yellow ‘not sure, or neither agree nor disagree’. This was a useful ‘member checking’ exercise in clarifying our understanding and establishing the degree of agreement on points made, and why some did not agree. Where there was disagreement, this is reflected in our report of findings below.

The sessions lasted between two and four hours and included breaks for drinks and food. All sessions ended with a ‘cool down’ activity led by our project partners from young carers organisations.

Data collection took place just as Covid-19 travel restrictions were easing; we adapted to the precautionary conditions that were felt to be appropriate in each site. All focus groups took place in person except one which took place online. There were about 6–8 participants in each group.

The ten interviews were carried out either in person, by phone or by video call. Interviews were semi-structured, in-depth and explored individual experiences of services.

### Analysis

The material for analysis consisted of the annotated flipcharts, notes written by both participants and researchers during the session, notes made by researchers after the session, and transcripts. The flipcharts included lists, compiled with participants during sessions, of the different services mentioned in each group, these were amalgamated and are presented below in Table 2.

**Table 2:** Services and support mentioned by focus group participants.

Service	Young carers (N focus groups in which mentioned, out of 19)	Parents (N focus groups in which mentioned, out of 2)	Total
**School and college-based support **			
Young carer Group/lead at school	6	0	6
Person at school/pastoral/SENCO	13	1	14
Time out room/card at school	10	0	10
Family engagement/support worker	4	1	5
Teachers	19	0	19
School counsellor	11	0	11
Other students	4	0	4
Free school meals/breakfast club	9	1	10
After school clubs/activities for young carer	4	1	5
‘Worry box’	1	0	1
School behaviour team	2	0	2
School nurse	4	0	4
Pupil premium	0	1	1
University lecturer	1	0	1
University counsellor/wellbeing services	2	0	2
**Young carers and carers groups **			
Young carers/carer centre groups and services	16	1	17
Parent Group by young carers organisation	0	1	1
**Mental health**			
Mental health worker/therapist for young carer	9	0	9
Mental health worker/therapist for care recipient	7	2	9
CAMHS for care recipient (sibling)	5	0	5
CAMHS for young carer	9	2	11
Family counsellor	1	0	1
Crisis team	1	0	1
**Social care and related **			
Social worker for care recipient	9	2	11
Paid carers	13	1	14
Council carers	1	0	1
Social worker for young carer	6	0	6
Family support worker	5	1	6
Family social worker	8	1	9
Social services	1	1	2
Multi-agency support team worker	4	1	5
Home adaptations/equipment	2	0	2
Respite carer	5	0	5
**Health and related **			
Hospital	5	0	5
GP/Doctor	8	2	10
Social prescriber	0	1	1
District nurse	4	0	4
Pharmacist	1	0	1
Occupational Therapist	2	1	3
Physiotherapist for young carer	1	0	1
Speech and language therapist for care recipient	1	0	1
Drug and alcohol support for care recipient	2	0	2
Autism services for care recipient	3	1	4
Autism/ADHD services for young carer	1	0	1
**Other sources of support/services in contact with **			
Family	16	0	16
Friends	11	0	11
Neighbours	4	0	4
Pets	2	0	2
Trips	8	0	8
Welfare benefits	5	1	6
Charities/Community support/volunteers	4	2	6
Clubs/activities for care recipient	3	0	3
Clubs/activities for young carer	10	1	11
Support at work for young carer	1	0	1
Case worker	1	0	1
Transport for care recipient	1	0	1
Helplines and online communities/support	4	1	5
Police	0	2	2
Adult learning	0	1	1
Peer support		1	1
Citizens Advice Bureau		1	1

Notes

1. SENCO = Special Educational Needs Coordinator; CAMHS = Child and Adolescent Mental Health Services; ADHD = Attention Deficit Hyperactivity Disorder

2. Frequencies are out of a total of 19 young carer focus groups and two parent care recipient focus groups

Notes relating to a single group or interview were added to a single file and entered into NVivo [39], so that the dataset consisted of 21 focus group files and eight interview files.

In the qualitative analysis we aimed to understand young carers’, and their parents’, experiences of services and support. The analysis focussed on seeking answers to the research question: what makes support helpful, or unhelpful, how can it be improved? We examined what was considered good or not good about existing services, and where it was felt improvements could be made. We took a reflexive thematic analysis approach, following the guidance of Braun and Clarke [40]. This approach was chosen because we wanted to be mindful of our role in interpreting the data, given our aim of learning from people whose voices are not often heard, and employing our learning to develop messages which would be of use to policy and practice [41, 42]. The approach allows for flexibility in the nature of themes developed and a combination of deductive and inductive coding. For the analysis presented in the current paper we followed the six phases set out by Braun and Clarke; they did not all take place sequentially. We familiarised ourselves with the data, by reading through and discussing fieldnotes, which included analytical thoughts made and discussed after each session, and noting insights and analytical ideas. We systematically coded the focus group and interview files using descriptors that were analytically meaningful in relation to our research questions. Next, development of initial themes was based on consideration of codes and discussion about meaning in the data; codes were grouped together so that the data relating to each candidate theme was collated. The presence or absence of particular aspects of support were at first grouped together (e.g. person was non-judgemental/person was judgemental), partly because views were not always easily categorised as positive or negative, but also because reviewing the different aspects together allowed a more nuanced analysis. Later stages could then pick out and separate recurring themes to do with what made a support helpful or less helpful. Thematically-coded material was reviewed both in relation to specific service types, and across service types, for more general messages about what aspects of support were valued, what made support valuable and appreciated, what made support unhelpful, or not wanted, and how existing support could be improved.

Techniques to enhance trustworthiness of the analysis included conducting the analysis iteratively, with the involvement of different researchers, paying attention to counter-examples and exceptions in the data and discussing our findings with our young carers advisory group and study steering group, and with relevant practitioners in a series of workshops; these were intended to ensure the integrity and relevance of findings.

### Ethics

Ethics approval for the focus groups and interviews was granted by LSE Ethics on 21 May 2021 (Ref. 1247). Informed consent or assent was sought from all participants and parental consent for children 15 or younger; consent and assent were discussed and given in advance of the groups and interviews, the voluntary nature of involvement was emphasised, both at the time of the initial discussion about participation and consent, and at the beginning of each session. Consent and assent were given in writing, except for online or phone interviews where verbal consent was given which was audio-recorded and noted on a paper copy of the consent form. The focus groups and interviews took place between 1/6/2021 and 31/5/2022.

## Results

Before setting out themes resulting from the qualitative analysis we first present tables describing the final sample of participants (Table 1) and listing the types of services which were mentioned and discussed by participants during the focus groups (Table 2).

### Sample description

As explained above, 133 young carers and 17 parent care recipients were participants in this research. Where known, information describing participants is presented in Table 1.

### Services spoken about by participants

Table 2 lists the services mentioned by participants in the focus groups. The labels reflect the language participants used, although some categories have been combined. The purpose of the table is to show the range of services and supports under discussion; the range is extensive, and some families were in contact with multiple services. Participants did not necessarily know the role or profession of the person they encountered, for example they were often unclear whether someone who visited them at home was a social worker or not.

### Themes from the qualitative analysis

The results of the qualitative analysis are now presented in two sections. The analysis resulted in some themes about what is helpful about what makes support helpful or unhelpful, and how it could be improved, that were specific to four particular types of service, while other themes were relevant across service types. First, we summarise the themes, and their characteristics in Tables 3 and 4. The following section then presents findings specifically relevant to the four service types: support for the care recipient; young carers groups; mental health support; and support in schools (summarised in Table 3).

**Table 3:** What is helpful or unhelpful about specific service types?.

Theme	Characteristics of theme
Service type: Support for the care recipient (care workers and other professionals visiting at home)	
Helpful support for the care recipient is valued by young carers but poor services can exacerbate burden	Can give the young carer a break/time for other things/less worry
	Emotional support and/or company for the care recipient gives them someone to talk to/share concerns with and can reduce the emotional load on the young carer
	Some caring jobs can sometimes be done better by a professional
	Practitioners can helpfully mobilise support from family, friends and neighbours
	Can cause stress, when new/untrusted, or too rushed
	Sometimes rude, unfriendly, don’t communicate effectively
**Service type: Young carers groups**	
Young carers groups are valued for providing understanding support, links with peers, fun and distraction, but do not suit everyone	Meeting other young carers, making friends
	Having fun, doing activities, time out, taking your mind off things
	Staff at young carers organisations understand your situation; there is access to an adult who can listen and understand
	Parent care recipients can also receive direct support through young carer organisations
	Some young carers may have concerns about leaving the care recipient, for longer/residential activities
	There can be feelings of anxiety or exclusion on first attending groups; groups are not for everyone
**Service type: Mental health services for the young carer**	
Mental health services are valued when you can talk to someone understanding and patient, but long waits and poor support can cause harm	Young carers value mental health support when they can talk to someone who listens and understands; provides flexible and patient support, and understands the young carer role
	Long wait times for support; often felt to not be worth the wait; CAMHS’ reputation puts people off seeking support
	Mental health supporters don’t always understand the caring role of young carers
**Service type: Support in schools**	
In school, understanding and flexibility are valued, but provision for young carers is variable	Young carers value the availability of someone to talk to (and a place to go)
	Time out cards can help a young carer leave class when need a break; early leaving cards are valued for supporting caring responsibilities
	Young carers value extra help and flexibility about schoolwork and homework; the right balance of wellbeing and academic support
	Schools are unhelpful when staff are not flexible or understanding
	Form filling for no apparent purpose is not appreciated

Note: CAMHS Child and Adolescent Mental Health Services

**Table 4:** Cross-service themes: What makes support helpful or unhelpful.

Theme	Characteristics of theme
**Finding, and linking to, other sources of support is valued, but not when it feels like being passed around**	Being linked with other services, appropriately, because the person listened
	Advice about, and help with, welfare benefits and housing issues
	Initial support in attending a service or meeting e.g. mental health support
	NOT being passed around services, ‘referred on’
	NOT having to constantly retell your story
**Listening and understanding are valued**	Someone to talk to
	Takes time, lets the person talk, and choose what to talk about, when they want to and are ready to
	Validates your feelings and experiences
	Adjusting support to meet specific needs and wishes of the individual or family; enabling activities that are important to them
	Social workers and other practitioners who don’t understand focus on the wrong things
**Trust, confidentiality and perception of risk affect how services are experienced**	Someone they get to know
	Someone who will not share what young carers have told them in confidence
	Awareness of young carers and the issues they face
	Bad experiences with, and reputations of, services result in deep mistrust of services (including fear of child protection intervention)
**Young carers value being involved in making plans and decisions**	Having the opportunity to ask questions and discuss solutions; both regarding services for themselves, and for the cared-for person
	Choice and flexibility
	Involving young carers can sometimes conflict with care recipient’s wishes
Practitioners need to find the right balance between being **proactive, persistent or intrusive**	Some young carers feel pressurised to be involved with/accept support for themselves, and can see it as ‘too much’ or ‘demanding’
	A balanced and flexible approach to individual and changing preferences and needs
	Holding contacts in places and ways that suit the young carer and family
	Being able to change your mind about wanting, or not wanting, support
There is **not enough support**	Some support would be helpful if it was more frequent/lasted longer
	It is unhelpful/distressing when services end with no warning

The subsequent section presents key themes about what makes support helpful or unhelpful which cut across service types. These cross-cutting themes are all important for the key service types presented in the first section, but as they are relevant across services we discuss them separately. These themes are summarised in Table 4.

Table 4 summarises the themes which were found to be broadly relevant across service types. The themes are discussed and illustrated in the second section of the findings.

The themes summarised in the above tables are explained below. Illustrative quotes are used to help explain the themes. These are often taken from researchers’ field notes (made both on the flipchart and elsewhere) and not always from transcripts, so are not always verbatim. As far as possible, quotes are identified according to the type of data collection (focus group or interview) and, where it is a young carer focus group, the age range of all those who took part in that group. All parents referred to are parent recipients of care by a young carer, their child. Some focus groups were specific for young carers of people with mental health or substance misuse problems, they are referred to below as Mental Health groups. Note that participants in other groups also cared for people with mental health and/or substance misuse problems.

## What is helpful or unhelpful about specific service types?

Four key service types were raised consistently as important: Support for the care recipient, young carers groups, mental health support and support at school. Table 3 summarises key themes related to these four types about what made support helpful or unhelpful. The themes are explained and illustrated below, with attention paid to where there were differing views among participants.

### Support for the care recipient

Young carers appreciated support for the person with care needs, from carers and/or other family and friends. This freed some of their time and helped to alleviate their worry for the person. One participant, for example, wanted the practitioner to focus more on the cared-for parent and less on herself (the young carer). Practical support and adaptations that made things easier for the person with care needs at home, such as rails, accessible showers or alarms, were appreciated. Some families had a certain amount of help from paid careworkers in the home, and a few young carers specifically commented that this gave them more time *‘it gives me a break when they’re there’*.

Respondents were not always clear what professionals’ roles were, but appreciated visits that gave the care recipient someone to talk to, to share concerns with, just for company, or as therapeutic input. As one participant commented: ‘*they calm him down*’.

Some of these people visited only once every few weeks but in other families paid careworkers came more regularly, taking on some of the regular caring and housework. This was reported as beneficial, not just for saving the young carer time, but also for the care recipient, because professionals could do some jobs better. One young carer described the paid careworker’s role as doing ‘*Pretty much everything else I don’t do*’ [Mental Health group]. Several carers were discussed in positive terms by both young carers and parent care recipients (*‘brilliant’*; ‘*really nice’*). Good paid careworkers listened to what was needed, and supported people’s independence, as in this example about help with bathing:

*They let me do the top bit so I feel like I*’*m getting my independence [Parent]*.

Many young carers in our sample would have liked this sort of support for the person they cared for but did not receive it, while some whose families received support would have liked more.

On the other hand, several participants reported bad experiences with support for their relative. Not all support provided to the cared-for person reduced worry for the young person. Unpredictable visit times were awkward and could provoke stress; for example, one young carer (13–19) was sometimes prevented from going to school when the carer did not turn up on time to relieve them. Another, who cared for a parent with mental health problems, said they (the young carer) often did not let the practitioner in when they turned up at ten minutes notice, because it was so unhelpful.

Young carers described lack of care, low standards, rudeness and unfriendliness:

*They are not very good, they don’t have a lot of training [young carer 13–19]*

*‘Council carers’. Always in a rush; we had to get rid of them. They were supposed to be helping get up in the morning and provide personal care but they weren’t nice. [young carer 16–25, from notes]*

Many respondents talked about changing or ‘getting rid of’ unhelpful careworkers. One young person reported that they themselves had higher standards than their parent (the care recipient), and that they pushed for better care on their parent’s behalf. Another had been told by their GP that they did not have a choice, they had to keep a careworker even though they felt the careworker did more harm than good because they did not speak to the care recipient [Mental health group]. Others were described as making a mess, or making mistakes, sometimes because of language or communication barriers.

Changes of careworker were more often not by choice however, and constantly changing personnel could add to young carers’ stress:

*If someone new is with my mum I get a funny feeling in my stomach if I leave the house as I don’t know if they will be okay [young carer 9–11]*

Conversely continuity can be reassuring:

*My daughter knows who’s coming in to help so she’s not edgy [Parent]*

One parent explained how valuable professional care support could be:

*If people see the effect by doing those ten hours a week for me and what it enabled me to do, it doesn’t just change my life, it changes my daughter’s [Parent]*

Valued support for the care recipient also came from family, friends and neighbours, and this could take some of the load off young carers’ shoulders, as this interviewee made clear when asked what helpful support they appreciated:

*I think probably my aunty taking her to her house, but even if it’s not that helpful, it’s just nice for her to be gone. [young carer interviewee]*

Paid services could play a role in increasing support from family, friends and neighbours:

*Now that I’ve got a social worker and all that the amount of jobs that I usually get have been cut a bit. Yeah because I just couldn’t handle the [school] work from the jobs on my shoulders. So I told [school SENCO (special educational needs coordinator)] about it and she put us on to a social worker and now my Dad’s doing a huge percentage of the jobs so I don’t have to do as much.*

This example shows how valuable support involves listening and understanding the situation, and that addressing it is not always about putting in more paid resources.

### Young carers groups

Young carers appreciated getting together with others who shared aspects of their experiences as carers. They also appreciated having fun, doing interesting activities, and having ‘time out’ from their day-to-day lives. Comments on attending young carers’ groups included: ‘*Fun’*, *‘trips’*, *‘socialising’*, *‘Gets me away from everything’*, *‘highlight of the week’*, *‘everyone’s friendly’* and sometimes ‘*you get food’*, *‘meeting other people like you*, *you’re not alone’*, *‘meet people in similar situations*, *who understand you’*, *‘Space to hang out with people like you’*. Occasionally there were opportunities for trips, which could address multiple needs as one participant explained:

*Residentials help you take time off and make new friends (that relate with your situation) and learn about the person you care for [young carer 16–25]*

However, some participants worried about leaving the person they cared for, when attending residentials:

*It was really good, but then some of the kids, they were like crying, because they weren’t sure whether the people who they were caring for were going to get any support [young carer 16–25].*

Concern about leaving the cared-for person was felt to potentially put young carers off staying away overnight. In this young adult carer group, participants concluded during the final sense-checking section of the session, that it would be good for extra care (which in some cases could be check-in phone calls) to be put in place while the young carer is away.

Important benefits of young carer groups included the availability of adult supporters for the young person to speak with, and being linked to other sources of support. Workers at young carers organisations were generally talked about in positive terms: ‘you can talk about everything’, ‘they know about young carers’. They were often described as listening and understanding, a theme discussed below.

Young carers groups provided different and multiple types of services to participants. There could be group-based or one-to-one, opportunities to share feelings and experiences, with workers, or with peers, as well as diverting activities, socialising and outings. Our young participants spoke about the support workers being helpful both for them and for the person they cared for. Some workers visited the family in their own home, or came to the child’s school.

Not all participants found all aspects of taking part in groups easy, however, especially at first. Some preferred the one-to-one meetings offered. A young person who had recently joined said: ‘getting used to new people can be tricky’, while another described it as ‘scary’. Others had not yet ‘plucked up the courage’ to attend a group, while a number who had opted not to attend groups that were offered described themselves as not enjoying meeting new people. Some participants commented that their group was good at helping if you struggle socially, while another said attending had really helped with their confidence. Some preferred to use spare time to meet with existing friends, but others mentioned having few friends, and appreciated the social aspects of groups. There can be issues in groups that make people uncomfortable, and a couple of participants who had attended a young adult carers group said they found it quite ‘excluding’, and that more could be done to involve new arrivals. Others found groups too noisy, ‘it gets quite out of hand sometimes’ [YC 12–16], too crowded, or the activities too restrictive. Some had had long waits to be given a place in a group, while in other localities, the young carers organisation managed demand in different ways, such as open-access regular drop-in groups, rather than waiting list. Demand also varied by area.

### Mental health support

While many participants expressed a need for mental health support, and some were receiving helpful support, a greater number reported that mental health support provided was unhelpful or not wanted. Many young carers referred to long periods on waiting lists to be seen (many months or even years); a common comment was that support was ‘*not worth the wait’*. Others said the support from CAMHS (Child and Adolescent Mental Health Services) was ‘*terrible’*, *‘always on their terms’*, *‘harmful’*, *‘tell you stuff you know already’* and ‘*limited*’. Several young people said they felt they needed mental health support but had been turned down by CAMHS, or were put off approaching CAMHS because of negative reports from peers, including, but not limited to, long waiting times. A parent commented that if a child was doing fine at school, it could be difficult to qualify for mental health support, while a young interviewee who had difficulty managing their anger felt support was stopped after three sessions because the issue was anger and not self-harm.

Participants in one of our young adult carer groups agreed CAMHS did not understand the impact of the caring role, and could be dismissive. They wished that mental health support could be more available to younger carers, to help them express their anxieties and stresses. Lack of understanding was thought to be the reason CAMHS workers sometimes made unhelpful suggestions. Examples given that were considered *‘ridiculous’* included: to take a bath when feeling sad (the young carer did not have a bath at home); to have a cup of tea; to do the washing up as a distraction from feeling anxious; not to cry or frown when feeling sad [young carers in one group aged 12–14]. Our participants often said that CAMHS provided support, signposting or advice which was not what was needed, and that this was because they were not properly listened to. Some respondents described CAMHS as being ‘*mean*’ or ‘*unpleasant*’ and that they could do more harm than good.

However, some people had received helpful support from CAMHS (‘fantastic’), as well as from other sources, including school and college counsellors, NHS adult mental health self-referral services for over-16s (previously IAPTs), and GPs. In counter to the point above, two young adult carers said CAMHS had specifically addressed their caring responsibilities and how they could be carried out. Helpful support from mental health services included proactive and flexible support, someone getting in touch when the school was aware of a family incident (e.g. health crisis for a parent); flexible arrangements to meet—for example at school, or in outdoor locations—and patience.

*They were like really helpful and my CBT [Cognitive Behavioural Therapy] worker, she took stuff at the pace how I wanted to go [young carer 16–25 re CAMHS Cognitive Behavioural Therapy].*

Positive comments often referred to the person being good to talk to, and this is explored further in themes below.

### Support in schools

Some schools seemed to provide better support than others. Perhaps because of an awareness of this, a school told one of our young carer participants they were happy to carry on offering them support even after they had left to go to another school. Young people often found a particular staff member easy and helpful to talk to; the most commonly-mentioned job roles of helpful staff were school counsellor, teacher, tutor, and designated young carers support person. The particular teachers that participants felt supported by were not necessarily ones who (still) taught the child but were ones where a relationship had developed and continued. This was valued.

*It was just I ended up having a good relationship with her ‘cos she was like always there when I needed to talk, like whether she was busy or not [young carer interviewee re a teacher].*

The way that support was provided varied widely. Support could be provided via regular support groups for young carers and/or as a drop-in service (sometimes with a therapy dog as an extra bonus). Some young carers were invited to check in with a support person at school every day; sometimes a support worker heard about issues/incidents through other avenues and would come to check on the young person. One participant told us they were often taken out of school for a walk and chat; some of our participants got understanding support from the ‘behaviour team’.

*My mum needs support because services say she overshares with me. Sometimes she’s upset and I don’t know what’s wrong and we end up arguing. I have someone I talk to at school twice a week. They are better than the last person I had, we didn’t get on. It doesn’t interrupt my lessons, it’s usually during form time [young carer 12–16].*

This quote shows that services for care recipients can and sometimes do take account of the carer’s needs as well as those of the care recipient, and suggests that there can be good coordination between services. Different approaches suited different people:

*At school there were one-to-one sessions but it just wasn’t the same–I (and others) really did not like coming out of lessons to go to a session, or out of break. Meant I missed time with friends. Not good. At the end of the school day would be better. At school, there’s never enough time, and there’s lots of cancellations [young carer 16–25, from notes].*

Sometimes there was a place, or a person, that the young carer could go to whenever needed, for example when feeling sad or angry. Our participants liked having a place to go in school, sometimes referred to as a ‘time out room’; sometimes (for primary age children) this was just a corner of a room. Some of these ‘places to go’ were nice places to be. Sometimes, given schools’ lack of space, the ‘place to go’ was also the ‘isolation room’, a term for the room where people went when they’d behaved badly. For some this meant it was a place they did not want to go; it could also mean there were other young people there, who had been sent there following bad behaviour, which some participants did not like. It was appreciated if there was an understanding staff member present in the room, but this was not always the case, sometimes there was no-one. Some young carers had ‘time out cards’ which they could show to teachers to be given permission to leave class. Although these were usually seen as a good thing, some participants commented that they were used inconsistently and not all teachers accepted them.

*Some people don’t know enough to be helpful (e.g. at school) [young carer 16–25]*

*It’s good if teachers all know their situation–if it can be on their record [it was in some cases]; teachers need to accept Time Out cards with no questions [young carer 16–25]*

One primary school had an ‘I’d like to talk’ box by the front door, where you could leave notes colour-coded to indicate the level of urgency. This was appreciated although some said it could be embarrassing when the staff member came to find them in class.

In our focus groups, some young carers noted that they, or their school, did not have the support available that they were hearing about from other participants in the group. However, even where there was this provision, not everyone who encountered the young carers at school was perceived as helpful. Some of the reasons for this are explored in the section below. Where feelings about school support were more negative, participants often felt school staff lacked knowledge about young people’s wellbeing in general, and prioritised discipline and grades over student wellbeing.

*There are two kinds of teachers–some just teach, others really care about children and understand the importance of wider wellbeing [young carer 16–25]*

*They’re given training on like if someone bumped their arm, but they’re not given training on like what to do if someone’s really struggling mentally, or how to talk to them about their family, or anything like that [young carer 12–18]*

In one college focus group it was considered that help was only available to people who ‘*make a fuss*’, or whose parents do. This had included our respondents in that group, but they didn’t think that was a fair system; they felt everyone should be equally supported.

There was help with schoolwork and homework; breakfast clubs were appreciated. Some young carers had passes to give them permission to leave school early, for example, to pick up a sibling:

*I’m ok, I don’t need anything, as long as my Mum’s alright. I have a pass at school that lets me leave at 2.30 to look after her but if I don’t have the pass I can’t leave. It’s stupid. [young carer 12–16]*

Unhelpful support in school included multiple form filling, which some participants described as feeling like the only goal of counselling sessions.

Although in general it was felt that mental health and wellbeing should be prioritised over academic work, there was the occasional mention of the converse view, that perhaps they could have been pushed more academically.

## Cross-service themes about what makes support helpful or unhelpful

Our data suggested strong themes about what makes support helpful or unhelpful. The factors set out below apply across services to people generally in a one-to-one role with the young carer or parent respondent, including social workers, family support workers and individuals in the service types discussed above. The themes are summarised above in Table 4.

### Finding and linking to other support, information and advice

People described as helpful were often those who, through listening and understanding, could find and get back to the young person or family member with information wanted, or were able to identify appropriate other sources of support, and help them with accessing and using that support. Young carers and, particularly, parents, commented that it was difficult to know what support was out there, what was available, without someone who could tell you about it. There was a strong feeling in one parents’ group, that opportunities were missed and that families came into contact with practitioners (e.g. in health services) who should have been able to inform the family about available support, but did not. Several young carers noted that it would have been very helpful for them to have been linked into support earlier, but they did not know it was available.

Social workers were among those in a position to supply sought-after information. One boy’s social worker was:

*Good because you could ask them to find out something and the next week they would get back to you with the answer [young carer 12–18]*

While an older participant in the same group had less positive experiences with social work:

*Need more and better info–get passed around, conflicting info, don’t know what’s available or what need [young carer 12–18]*

Parents made similar comments; A ‘good’ social worker was able to access support, for example regarding children’s special needs and associated support, holiday schemes and respite for children. Social workers whose support was appreciated helped intervene with landlords, getting housing adaptations in place, and sometimes acted as an advocate to get things done. They could also help young people defend themselves: ‘She used to go to meetings with us, just to basically say how well I was doing.’ One young carer of a sibling with autism noted it had been helpful to be linked to other families facing similar difficulties.

In one college-based focus group, participants felt support at college was more useful than support they had received at school specifically because it did help with outside-college issues. However, some school-age participants in other groups also received support for problems outside school. Important information, from social workers and others included informing people about government benefits and how to get them:

*Having like the carers allowance, or things like that, like that sort of stuff just isn’t really talked about and then it’s hard to find [by] yourself [young carer 12–18]*

The quote suggests a progression, the need to know that the benefit exists, that you may be entitled to it, and how to get it. Money problems raised included: reduction in carer’s allowance once the child turned 18; being turned down for carer’s allowance because another family member was already claiming it (which is not supposed to be the criterion, as it is supposed to be based on how many hours you care for), and being turned down for Personal Independence Payments (PIP), then overturned on appeal. There were also some positive comments about PIP assessments.

Linking people to other support had to be done sensitively, however. There was a strong distaste in many cases to being ‘referred on’, when it meant someone new getting in touch, asking you to tell your story again, sometimes when you didn’t know what organisation they were from, why or how they knew about you, and what if anything they were offering. A few individuals described positive experiences of having a practitioner (such as from a young carers organisation) who would support you in attending another service (a mental health service for example) for the first time. Conversely, a referral with no follow-up from the referrer to see what had happened seemed to indicate a lack of care as in this discussion about “bad social workers”:

*The ones who can’t be bothered to take out some of their own time—they’ve got to make sure like the person that they’re supposed to be helping is actually getting help, so they’re just doing the basic minimum … as long as you’re still getting like your pay cheque. [young carer 12–16]*

Here, the ‘bad’ social worker is framed as doing the minimum required to keep their job, rather than being motivated by providing helpful support. How linking to useful support could be done sensitively and with the involvement and understanding of the young person or family is related to the themes set out below.

### Listening and understanding

Having an adult to turn to and talk to was repeatedly raised as a feature of good service provision. While many participants were happy to discuss their caring situation with peers, others preferred only to speak about that with adults. Being someone the young person wanted to open up to did not seem straightforward, however, and we had many accounts of the young person saying they had not wanted to talk or be questioned, in particular circumstances, or sometimes as a general rule. These issues are a central theme cutting across service interactions. Someone the young person felt they could talk to was highly valued:

*I pretty much rely on those kinds of people because I can’t talk to my mum about that kind of stuff [young carer 9–12].*

People in various roles with respect to the young person could take the role of ‘someone to talk to’, the most commonly-mentioned were school staff and young carers’ workers from groups in or outside school. Other people taking this role included social workers (‘we do fun stuff, it calms me down’ [young carer 12–14]), family workers (‘easy to talk to’ [young carer 12–14]), paid careworkers who came to the home, GPs, family members, friends of the family, and neighbours. Participants had different preferences and opinions as to whether they liked to see someone that was there for the whole family or just for them, and young carers also often said they wanted someone for the cared-for person to talk to.

Many young carers lived in complicated family situations and many had, or their parents had had, poor experiences with services in the past. Some families had conflicting perceptions of needs for support, and roles, between individuals in the family. A strong theme across all groups and most interviews was that a feature of helpful and appreciated support was when someone listened to them and understood them. This was a feature of appreciated support from services including schools, social work, and young carers organisations.

Whether or not a particular worker was good at listening, understanding and being helpful was often said to be ‘*down to the individual’*. This could be because of personality, experience, skills, or the amount of effort the worker put in, or time they had. Descriptions of helpful support were often set against descriptions of what had been unhelpful. In one young carer focus group’s discussion about ‘*good social workers’* and ‘*bad social workers’* the spiralling effect of poor support was described as being due to:

*Just overall lack of communication. The social worker doesn’t take enough care in exactly what that person needs, and then because of that the young person doesn’t want to communicate, because there’s no trust, and then the communication falls down, and then nothing happens… [young carer 12–16].*

Good listening was described in different ways: you can ‘*talk about what you want to talk about’*; they ‘*listen to you*, *complement you’* (both referring to young carers support workers) ‘lets me speak’ (regarding a young carers worker at primary school). In another group, specifically for young people who cared for someone with mental ill health or substance misuse, a social worker was described as being ‘*a good one’* because they allowed you to speak about issues about the cared-for person, and validated these feelings and experiences ‘*allows you to be angry’* and ‘*discusses facts*: *“I know your mum does this*, *I’m annoyed with her too”‘*.

However, ‘*lets me speak’* was different from feeling obliged to do so:

*Some are nice and listen, others aren’t so good, make you take the lead in conversation, which is difficult [young carer re CAMHS]*

It is a feature throughout these results that different approaches work for different people, leading to a conclusion that person-centred, flexible and listening approaches are needed. Good support was described as changing based on having listened to what the respondent said. This could include, decisions on where and when contacts would take place, it could include providing access or equipment that might enable someone to do something that made a difference to them. One participant said a support worker was trying to source a bicycle for them, for example, and another had been helped to acquire a laptop. Choice and flexibility about what was offered, for example at a young carers centre, was valued: ‘Gives you things to do, but they’re optional’.

A lack of listening was linked to less helpful support. One social worker ‘*doesn’t always pay attention’*, while another ‘*makes decisions for you*, *doesn’t listen’*. Speaking to someone who did understand was described as ‘*a relief’*. However, some practitioners were described as listening, but not understanding. Some participants described being upset by being asked to explain more, and feeling unable to; there was a common theme about a lack of understanding about the young carers’ situation.

Young carers sometimes had a wealth of experience both of services, and of the person they cared for. Not being taken seriously led to not being understood. For example, one young person felt that preventative action could have been taken if the family’s raising of concerns had been taken seriously by the first GP they had been trying to access support through. Lack of understanding was also linked to lack of knowledge or consideration of the issue.

Young people caring for a parent with a mental health or substance misuse problem often felt their role and its impacts were dismissed:

*You’re not an official/professional carer so you’re not supported… The role is not acknowledged [young carer 16–25]*

This young person felt he was doing ‘therapy’-type support with his mother, she spoke to him about difficult things, but he wasn’t given any support to deal with it.

### Trust, confidentiality and perception of risk

Many young carers, as well as parents, referred to a ‘good’ support person being someone they could trust, and a ‘bad’ support person being someone they could not trust. Many children, young people and parents in the study were distrustful of services, and had deep reservations about talking to people outside the family.

*If they come to see you and you don’t know them they could use the information against you [young carer 9–11]*.

*I wouldn’t talk if they came home because they can search you up [young carer 9–11].*

Young carers described some visits as ‘*intimidating*’ or ‘*concerning’*; identifying the visits as connected to child protection procedures. However, this theme was more likely to be explicitly related to fear of child protection intervention in parents groups. Parent participants talked of feeling frightened of social workers because they have power to take your children away, and this could also lead to feeling frightened of seeking help in general:

*Advice needs to be available somewhere where you don’t feel in danger of your child being taken away [Parent]*

*Feels like the system is there to see when you fail; you think someone is there to help you, but then you see they are keeping tabs, waiting for you to slip up [Parent]*

Sometimes, trusting someone was down to having someone the family, or the individual, had got to know. Participants who were reluctant to share their stories with services said that talking to someone outside the family would be ok if it was ‘*someone you knew really well’* [young carer 9–11].

Young carers in our study often had positive things to say about individual social workers (parents less so) often, again, contrasted with less helpful support in that role.

*‘I think the helpful ones always leave too soon, but the ones that don’t know anything stay way too long.’ [young carer 12–16]*

Participants who had difficulty trusting practitioners sometimes developed a bond with a particular person; these could be valuable relationships, but often, systems in place meant that when you made progress (for example improved mental health) the relationship was terminated. One young carer described this process in relation to a therapist with whom contact had ceased following her improved mental state; she was very sad about the contact ending and missed her therapist.

### Breaches of confidentiality and sharing of information/awareness

Many participants gave a negative rating to a school staff member or a social or family worker because of breaches of confidentiality:

*Can’t trust them, they will tell the head and get you referred [young carer, mental health group re school tutor]*

Here, being referred is presented as a negative outcome, speaking to the theme of risk perception.

*Not trustworthy. You put trust in them and then they tell other teachers and don’t help [young carer 11–15 re class teacher].*

A perceived breach of confidentiality could lead to disengagement from services:

*They told other people when I didn’t want them to [young carer 12–16 re social worker].*

As a result of one breach of confidentiality, the young person stopped communicating with the social worker. Conversely, young carers in one group were very positive about a particular school counsellor and agreed:

*You can trust her; [but] teachers will tell other people/other teachers [young carer 9–11].*

But teachers could also be a trusted person:

*Won’t go ‘round telling everyone’ [young carer mental health group re class teacher]*.

*Conversely, sharing of information could be fine, if it was discussed with the young person first. A positive rating was given to a member of school staff who:*

*Uses the info you give her but not until/unless she needs to and it’s in private [young carer 9–11].*

Examples of good practice given by young carer participants were when the staff asked if it was alright to share aspects of conversations they had had with the young carer’s parent; or when the practitioner had ideas of other useful referrals/connections, but checked with the young carer before going ahead.

While there was a strong desire for confidentiality, young people also appreciated if there was a certain level of awareness of their situation, if this awareness was combined with understanding. At school or college, where this could support helpful interactions and allowances:

*I would like college to know what I am going through a bit more… Only my main college tutor knows, and she allows me to leave things earlier [young carer interviewee].*

Conversely:

*School can be a bit of lottery though, I had one maths tutor that just didn’t get that I was a young carer, I got really stressed and stopped going to his class. I am having to take extra maths classes now.*

There were mixed feelings about how much awareness young carers wanted other people to have about their caring situation; some reacted strongly against the idea of some other people knowing (the example of a Scouts group was given, and some school staff); this seemed to be partly because there were some spheres where they just didn’t want to have that persona, or have to talk about it, but in other cases it was because they did not like people to know about their young carer status if they did not understand it.

It is personal and I don’t want to share if people keep asking [young carer 12–16].

Young carers did not want people to know personal details of their lives, when they had not shared it themselves, or had not approved the sharing of the information, but they did want people to be understanding of some of the repercussions, for example, to do with attendance, homework, or, sometimes, behaviour or becoming upset.

As we’ve seen, participants were keen that there should be greater understanding of young carers; they felt there was poor understanding of what was involved and that there should be better understanding in professions such as teaching, the police, nurses and other health staff. To some extent the sharing of information about individual cases could help support this, but this would need to be done through discussion with the individuals involved about what was appropriate and useful to share.

### Involving the young carer in making plans and decisions

A theme which intersects with the appreciation of practitioners who listen, understand, and can be trusted, is that of involving the young carer in decisions that affect both them and the people they care for. Both young carers and parents, unsurprisingly, appreciated it when decisions about referrals concerning them were discussed with them. They also appreciated it, when they wanted to know more about the cared-for person’s condition and supports, if these were discussed with them.

Frequent comments from participants related to people getting in touch and decisions being made, that they did not feel they had been involved in:

*When younger than sixteen he really felt he had been shut out of care, even though he was caring. He wasn’t consulted or included in discussions [research notes re young sibling carer, 16–25].*

Participants appreciated having their role taken into account by services supporting the care recipient. This was mentioned, for example, in relation to positive ratings for an assessment for PIP, a GP, and a pharmacist. Young carers appreciated being kept informed about changes in medication and care for the care recipient, and having these explained.

Young carers of siblings with care needs, where there was also a parent providing care, were especially likely to feel undervalued. One participant felt that he was not allowed to provide care in the way he wanted. His sister, with autism, had activities arranged by their social worker, but he wasn’t included in these. He felt that, for many activities she would get more from the experience if he went with her, that she would participate better and feel less anxious. He told us that his attendance was never thought of, even for something like the cinema, and usually activities were arranged without him.

A number of participant young carers voiced frustration at having information about the care recipient’s condition withheld from them (with confidentiality sometimes cited as the reason), or of professionals taking the (parent) care recipients’ account as the correct one, without listening to the young carer’s version. Conversely, another participant, whose sister had special needs, was encouraged to ask questions by the service working with her. This was appreciated. One young person knew that her mother, whom she helped care for, preferred her not to be too involved, and that because of this her mother withheld information which the young person would rather be informed about. This young carer’s conclusion was that it would be better for a professional to take the roles her mother was uncomfortable about the young carer helping with (in this case, taking medication). Another young carer objected to being told to go upstairs and stay out of the way whenever a person came to help her mother.

An important aspect of involving the young carer relates to service professionals for the care recipient considering the wellbeing of the young carer. But several young carers described services which could have taken their role and needs into account but did not. Social workers and care workers (at least sometimes identified as being for the care recipient) were often identified as being ‘*dismissive*’ of the young carer. This was agreed by participants in a young adult carer group:

*Good ones will help get you through stuff, most only think of the person they care for [young carer 16–25].*

Young adult carers in one group made involvement of young carers a key message for government if they want to improve support for young carers:

*Listen to young carers and recognise when they need help and help them! [young carer 16–25]*

We’re the best judges of our needs, ask us instead of trying to guess and f**ing it up! [young carer 16–25]

### Proactive, persistent or intrusive; changing your mind

In this section we focus on a theme to which many of those above are related–the difficulty of matching the level of persistence from services to individual needs and feelings–finding the right level of proactiveness, without being intrusive. As this level could be different for each family or each person, services need to be flexible in their approach.

In a focus group for young carers of people with mental health conditions, a helpful social worker was described as one who does not make demands, fits around the family rather than the reverse; doesn’t tell them what they have to do, and is flexible about visit times. Appointments made at inconvenient times could cause problems and stress and it was not appreciated when people appeared to turn up ‘uninvited’ or ‘randomly’. However, there were differences of opinion on this point in another group. While some said that people who do not say when they’re coming is unhelpful, others felt that random visits could help show services what was really going on at home [young carer, mental health group 12–18]. Some of these ‘*random*’ visits may have been stipulated as part of child protection or child in need plans.

### Flexibility in approach/person-centred/on-call or calls you

A theme across several focus groups was of support that was ‘*helpful*, *but sometimes too much’*. *‘Too much’* here could mean too intense, too persistent, too pushy or goes on too long. In one group most participants agreed that they kept being offered help they didn’t need.

*Good that they help, but it’s sometimes too much–you don’t need help but it doesn’t stop [young carer mental health group 9–18 re social worker].*

One described support at a young carers centre as ‘*good*, *but sometimes too much’*. Another interviewee who had recently attended a group (online) for the first time, described how nervous they’d been. She said:

*But they, like, encourage you a lot to do it, and it’s a bit pressuring [young carer interviewee, re young carers centre]*

In another group two young carers made a similar point, that a social worker, or family worker, was ‘*Too demanding’*, they kept referring you to people you don’t want to go to and/or were too tired after school [young carer mental health group 12–18]. Some said they had been referred to things they didn’t want and one participant said he’d been referred to something even after saying no [young carer mental health group 12–18].

Parents also sometimes described support as ‘*too much’*. A parent commented that support could be:

*Difficult to get rid of if don’t suit you, if it isn’t working. Formal complaints sometimes work, sometimes don’t (but I don’t actually want to go down the complaints route) [Parent]*

Other comments from young people showed that some workers trod this line between being proactive or over-persistent carefully. Some groups provided opportunities for one-to-one chats within the group setting, which was valued. One young person [9–11] said how important they thought it was that the worker checked in with them personally to see how they are doing with the group.

Workers from some young carers centres would regularly call round to check on people, which could be appreciated:

*When she’s on the phone she always says, do you have anything that you need to speak about? And like if it’s not that, she just like rings every so often to check up, and she offers like me to go to either a one-on-one session or like a group session, to discuss like how I’m feeling, and things like that, just vent really. [young carer interviewee].*

This young person had not yet taken up the offer of one-to-one support but appreciated that someone was checking in. This was a repeated theme. Providing flexible support that could meet young carers’ changing circumstances and feelings sometimes involved this ‘checking in’ while at other times the option of making contact was given to the young person. Some young carers valued having a number to call if needed.

This idea of ‘on-call support’ was discussed in several groups, often as something that was lacking:

*There’s no support in between crises [Parent]*

A few people did feel they have some sort of support person they could call when needed, while others said they would call emergency services if there was a crisis.

#### Opportunities to change your mind

We have seen that having an adult to talk to was highly valued by young carers, and that this needed to be someone who could listen and understand, and could be trusted.

Getting the level of proactivity or persistence right enabled people to be able to change their mind about receiving support. There were several accounts of services offered being turned down, either by the young carer, or by the care recipient. One young person described turning down support because they did not see themselves as a carer, but they were given the opportunity to change their mind later, which they did. A teenage young carer told us that the support workers were helpful but the young carer didn’t feel they could open up to the support worker, leading the worker to conclude the young person didn’t need them, when they felt they did.

Conversely another young carer interviewee felt they did have the option to change their mind, and could go back to their contact at the young carers organisation when they needed to:

*I think it’s just like the offer–the support–even if I decide I don’t need it right now, it’s always an option. Like they said, if you ever do need us, just contact us. And I think that’s really good cos they don’t think, oh well they don’t need it, so they disregard it completely. It’s always an option. [young carer interviewee]*

There were examples of both care recipients and young carers changing their mind about wanting a carer to come to the house, being initially reluctant.

*He assessed me, I got a package, and then I felt embarrassed… and so I stopped the package, and then they referred me this year… I’ve hit myself in the head, do you know what I mean? I’ve like give myself a wobble and said look, it’s not fair on your children [Parent].*

This parent eventually restarted the support offered, despite her reluctance, seeing the potential benefit for her children.

The opportunity to revisit a previous ‘no’ needs to be available, at the same time as not overdoing repeated contacts that some young people, as well as some parents, objected to as over-persistent or intrusive. In one group [young carers age 16–18] the problem was summarised as staff being unavailable but, conversely, that they won’t stop giving support/contacting you when you no longer want it.

### Not enough support

Although we have seen that ‘support’ was not always appreciated, that sometimes support was offered when it was not felt needed, and that it could be ‘too much’, a huge theme in the analysis was that there was not enough of the support people appreciated. When asked how support could be improved the answer was often that there needed to be more of it.

Young carers groups and activities for example should be available more often (one young person whose group ran once per fortnight felt this wasn’t enough), others: ‘*would like to be able to do this more often*’ [young carer 12–16] ‘*a bit more often and more activities’* [young carer 12–16]. And even during the group:

*There’s not always enough support in young carers [groups] because there is so many young carers [young carer 12–16]*

Sometimes, even where a worker had identified the needed support, service constraints prevented this being put in place or continuing. One parent reported having been told they should have had family support but because of staff redundancies the family received what was described as ‘Child Protection’ intervention instead. A young adult carer noted that a social worker used to take the care recipient out, realising this was the best help that could be given, but the social worker was taken off their case, allegedly for being ‘*too involved’* [young carer 16–25].

There were many comments regarding support at school and college along the lines of workers not having enough time:

*Helpful but too busy [re. school support person, young carer 9–12]*

*They are very accomplished but can be very busy [young carer 13–19]*

There was an awareness of stretched resources:

*So many teachers just don’t have the time. I mean like a Head of Year is looking after like what, 700 kids [young carer 12–18]*

And similarly for family support and social workers:

*Really nice, tries to help, makes me feels better, I would like to see her more [young carer mental health group 9–18, re social worker who visited school twice per week]*

*Good when do come but could contact more [young carer 12–16]*.

*Could be improved if they stayed longer [young carer 9–12 re social worker for mother]*.

*Helped with mum when feeling down but only stayed 3 weeks [young carer 9–12 re family support worker].*

Support often ended before the person receiving it wanted it to. Sometimes the ending did not appear to have been warned of in advance and endings could happen because of seemingly arbitrary service boundaries. Parents in one group talked about service silos leading to lack of accessible and sufficient support, they were constantly being told by services that it is ‘not their issue’ and that the onus is on parents to navigate services.

‘Not enough’ could also mean not enough at the right time: support sometimes came too late and was not then welcome; the opportunity to talk to someone needed to be there at the time the young person wanted to talk; and/or waiting lists could mean participants did not get help at the time they needed it.

These service-level issues contribute to ‘not enough’ support being received, and underline the importance of a practitioner who will help people to navigate services, as discussed in the ‘finding and linking’ theme above.

## Discussion

In this study we spoke to many different young carers in four contrasting areas of England to explore their experiences of services and supports. Our analysis developed a range of themes about what good support looks like, and why some support is, conversely, experienced as unhelpful. We addressed a research gap in how best to support young carers, from the perspectives of young carers themselves, in the current service environment in England following increased legal rights for carers. A feature of current services which was not as developed at the time of previous research is the presence of voluntary sector young carers organisations [4], some of which facilitated our research. Young carers organisations were a frequent service and support topic, likely reflecting that we often contacted participants via these services. Addressing the research gap through a qualitative analysis has led to a range of novel findings, explained above, including some specific to particular service types, and others which are relevant across services; these are summarised in Tables 3 and 4 above. Key initial points arising in focus groups were ‘member-checked’ with participants in groups, at the end of each session, and the final themes developed from the analysis were discussed with, and validated by, young carers and with practitioners in our advisory groups and at dissemination events to assure relevance, and resonance with their experiences.

We found many examples of positive experiences with individual practitioners. Some young carers had experienced what good support looked like and could point to key services that were helpful (support for the care recipient, young carers groups, mental health support, and support in schools). For all these categories of services there were negative experiences as well as positive ones. Young carer participants could explain what made a support helpful including someone who listened and understood, could be trusted, could support them to access the right kind of support or activities, and/or would involve them in decision-making. The findings show that needs expressed in older research for more and better services for the cared-for person, and more opportunity for breaks from caring [16, 43], remain highly valued, but are often lacking in both quality and quantity. Our research suggests that this remains the case despite new rights in law, stating that young carers should not be providing care that poses a risk to their wellbeing, education or life chances [44;Subsection 2.50]. That these risks to wellbeing exist has been demonstrated elsewhere [4, 14] as has the ‘failure of implementation’ of the legislation thus far in identifying and assessing the needs of young carers [45]^,^. A contribution of our research is showing that previously identified weaknesses in provision remain, despite extensive policy and practice development. Failures of provision can be because no support is provided and/or because existing support needs to be improved. A further major contribution of our research is that it uncovers specific factors which make existing support helpful or unhelpful from young carers’ perspectives, so giving valuable indications of how existing support can be improved.

In our study we found a strong theme about wanting more support, provided services being too infrequent, or services ending too soon. It may well be, as people running such groups have suggested, that young people end up in a young carers group because of its relative availability, rather than because it is the type of support they most need. Young carers groups in and out of school provided peer support, shown elsewhere to be valuable [46], as well as support from adults. Young carers groups also face funding difficulties however, in line with the general decline in voluntary sector funding since 2010 [47].

But there was also, importantly, a central theme in our findings that much support is not welcome because services are not trusted, or don’t listen or understand. This lack of trust has been found in other research, particularly in relation to social workers, and has been suggested to have been exacerbated by increasing focus on safeguarding over wellbeing [48, 49]. Young carers and parent recipients of care in our study reported many negative behaviours from practitioners towards them, including criticism, rudeness, breaking of confidentiality and being passed around services. The many examples of support *not* being appreciated may suggest that there is at least some scope for improvements to services for young carers and their families within current budgets.

However, getting support right for families with often complex difficulties, and sometimes conflicting feelings and wishes, is difficult. Our findings suggest that while key features are commonly agreed on by our participants, exactly how listening, trustful interactions can be achieved may vary. We present here some key factors arising from our analysis which could be seen as opposing:

- Awareness versus confidentiality: some young people wanted those around them to be informed about their caring situation and its implications, while others wanted these to be kept secret.
- Proactive or intrusive: some young people felt a support person should be getting in touch with young carers regularly rather than leaving it up to them to get in touch, while others found this intrusive
- Linking in or handing off: some interactions were seen as dismissive if the young person felt pushed off to another service where they had to tell their story again, while others appreciated appropriate referrals or introductions

These opposing factors, or factors perhaps lying on a continuum, underline the importance of a person-centred, listening and flexible approach. In such an approach involving the young carer in decisions and plans which affect them seems crucial. Where the right spot is on these spectra will fluctuate for each young carer and over time, as life, feelings and caring situations change. Practitioners need to make decisions about what the right fit is for each person, and at each time point. The Care Act (2014) allows for, and supports, such flexibility. Our findings from young carers can help inform further efforts to implement these aims in practice. Our participants described good practice as someone taking the time to listen and understand, and thus find out what is important to the family and its members and what can best be put in place to improve their quality of life. The dilemmas over confidentiality often seemed as though they could be addressed through discussion with the young person over what they felt should be shared, and with who. In schools, while acknowledging the demands on school staff, more discussion about the issue of young carers could support more sensitive reactions to things like lateness or missing homework; our young people described many positive initiatives in some schools, but others where this was lacking. Dilemmas about the level of intrusion can be negotiated and agreed (young carers can be asked, for example: when would it be ok for me to give you another call?). There are skills and resources implications, of course, in making those extra efforts: talking and listening to families about what is the help they want and need, and what is the help they don’t want. However, in the long run, appropriate and appreciated person-centred support could prevent future problems and negative impacts of caring, and therefore be cost-saving [14].

Services are more effective and efficient when they are a better fit for individuals and many of the themes we have set out speak to advice coming from strengths-based approaches such as: looking at the family and other networks to see what support can be mobilised; developing a trusting relationship between a practitioner and the person seeking support; understanding what is important to the person (e.g. activities, interests) and linking in to other support available in the community and elsewhere [50, 51]. We saw that people’s situation needed to be understood to get support right–and this might mean putting in paid care, but it might also mean intervening to get family members to consider roles and responsibilities (who in the family and broader social network could be doing more, or doing something different, to make life easier for the young carer and the care recipient?). It might mean finding out what is important to that individual–what activities or hobbies for example might be the thing that makes their life feel good.

Services will be most effective, and cost-effective if they can intervene at a point when they can prevent problems developing or getting worse. A key consideration for making support available at a time when it could prevent future crises and/or poor outcomes further down the line, is taking advantage of opportunities to find out if support is needed; a number of the themes above relate to missed opportunities to intervene; ‘involving the young carer’ encompasses services for the cared for person taking into account the role, and needs, of young people in the same household. Young people, and those they care for, need sensitively handled opportunities to change their mind about accepting support.

This practice is not easy, it involves skill, compassion, knowledge, understanding, and time [49, 52]. How can systems support these approaches? Relationship-based and strengths-based approaches aim to change the focus of services, away from managerialism towards more trust towards frontline workers to try creative, person-centred ideas [50, 53]. System-level issues are raised by young carers, for example: waiting lists; opportunities to change your mind; checking in; service boundaries and silos; referral processes; but what of the more individual-level factors? Do system-level issues also need to be addressed to allow for the type of support people want to be available? We heard that practitioners being too involved with a family could be seen as a failing by management and that activities which made the biggest difference to families (e.g. supporting the person with care needs to go out for a couple of hours) were not always considered appropriate uses of time. It has been suggested that a truly ‘strengths-based’ approach requires whole system change to the way support is provided, shifting attitudes and priorities of both those receiving and delivering services towards greater collaboration [54]

The range of different services discussed in our field work (Table 2) is evidence of the complicated system within which families may be needing, seeking and/or receiving support, and within which practitioners are working. Systems theory has been used to understand and examine complex interventions, and how to evaluate them, and has been applied to service environments such as those we are discussing here, where multiple services (for example social work, education, mental health and voluntary sector organisations) and multiple individuals may be involved and affected. Brimblecombe and colleagues have emphasised the importance of taking a carer-inclusive approach when thinking about the unmet needs of individuals [55], while also considering the broader contexts which place constraints on the actions of all members of what has been termed the ‘care triad’ of care recipient, unpaid carer and practitioner: a contextual triadic approach [38]. These contexts include front-line practitioners in contact with families, local authorities, and national government; provision of care is ‘rationed’, despite policy support for meeting needs, at all these different contextual levels, as Arksey has argued [56].

Taking the time to listen to young carers and care recipients can help identify barriers to seeking or using support, or to support being effective. Helpful support is tailored to the particular contexts surrounding an individual, family and community. ‘Systems thinking’ can help avoid siloed thinking and encourage thinking about how a particular site or method of intervention can interact with wider systems [57]. Thinking about support aimed at a particular person or problem (for example the care recipient’s difficulties with daily living) as part of a broader network of influences can help prevent unintended negative consequences to other parts of the system (for example, the young carer’s education or wellbeing).

The positive features we identified show that it is possible to offer support to families in a supportive, unthreatening and more listening way, but that negative experiences, and negative attitudes by young carers and/or their families particularly towards social services, are still common. Fear of involvement and distrust of services have been demonstrated elsewhere [27, 49, 58] and suggestions to address this have included greater separation of child protection and family support services, though this carries its own risks. Good relationships between those needing/seeking and providing support are key, so attention needs to be given to the factors that get in the way of productive relationships (including lack of practice resources and staff turnover), and factors that promote good relationships (listening, taking time to understand, trying things out, linking effectively to other available support). Some of these factors reinforce findings from previous research such as the difficulty of finding out what support is available [59], and appreciation when information about the care recipient’s condition is shared [46, 60].

We set out to include underrepresented groups in our study and were successful in recruiting young people who cared for people with more stigmatised conditions, such as mental ill health, and those relatively more isolated including young carers in rural locations and those who did not attend any support groups. Additional challenges were created by the Covid-19 pandemic, and we were not able to include young people from the Roma and Traveller communities, or asylum seeking or migrant young carers, where particular problems with access to or engagement with services have been identified [61]. These missing voices could fruitfully be the focus of future research. A great deal of pre-recruitment work will be needed to develop trusting relationships with marginalised communities, work that was not possible with pandemic restrictions. Restrictions on recruitment activities meant we mainly recruited via young carers groups and the participants have therefore been identified as in need of this support. As young carers are often referred to services via their schools, our participants may therefore attend schools that are, on average better informed. While this can be considered a limitation, for the purposes of this analysis, it could also be considered a strength, as we were keen to identify what can make a service or support acceptable and helpful. Our study focuses on the English context where rights and support for young carers is seen as relatively well-developed [9]. However, young carers exist worldwide and our study has relevance to those planning, developing and delivering support for young carers in other countries, bearing in mind different legislative and cultural contexts.

Future research, building on our findings, could usefully explore the variation in practice approaches and factors supporting successful assessment and implementation of the needs and strengths of families where there is a young carer. Given the complexity of the service environment, mixed method approaches are likely to be needed [62].

### Conclusion

We have identified many ways in which young carers found support to be helpful, and ways support could be improved. Many of these aspects of valued support are already represented in legislation in England–the 2014 Care Act [63] and the 2014 Children and Families Act [64], but are not yet consistently implemented in practice. There is scope for optimism in our findings, given the identification of positive support, and potentially some scope to make better use of existing resources, given the potential waste of resources associated with negative experiences with services. The message is not just that more support is needed but that support could be provided differently to make better use of resources. Here we have shown that good support does exist, but that not all funded support is beneficial. Support needs to be flexible and adaptable, and people need to be given additional chances to accept or make the best of support. Sharing of positive experiences can be beneficial for both people seeking support and those delivering it, and this paper has sought to present what we have found about what can make support valuable. Together with our young people’s advisory group, we are working to share these messages with young carers, their families, and the broad range of services who are in a position to support them.
